# Herbal Weight Loss Supplements Induce Metabolomic In Vitro Changes Indicative of Oxidative Stress

**DOI:** 10.3390/metabo15090587

**Published:** 2025-09-01

**Authors:** Emily C. Davies, Garth L. Maker, Ian F. Musgrave, Samantha Lodge

**Affiliations:** 1 Centre for Computational and Systems Medicine, Murdoch University, Perth, WA 6150, Australia; emily.davies@murdoch.edu.au; 2 Medical, Molecular and Forensic Sciences, Murdoch University, 90 South Street, Murdoch, WA 6150, Australia; 3 Adelaide Medical School, The University of Adelaide, Adelaide, SA 5005, Australia; ian.musgrave@adelaide.edu.au; 4 Australian National Phenome Centre, Health Futures Institute, Murdoch University, Harry Perkins Building, Perth, WA 6150, Australia

**Keywords:** herbal weight loss supplements, ^1^H NMR spectroscopy, HepG2 cells, Caco-2 Cell, metabolomics, oxidative stress

## Abstract

**Background/Objectives**: The prevalence of obesity continues to rise globally, and with this an increase in the use of herbal weight loss supplements (WLS). At present, there is limited evidence to support the efficacy and safety of WLS, and there have been growing reports of adverse events associated with their use. We aimed to determine those WLS that caused toxicity in vitro and to use ^1^H nuclear magnetic spectroscopy (NMR) to examine the metabolomic changes induced by these WLS in human hepatic and intestinal cells. **Materials and Methods**: This study used in vitro methods and ^1^H NMR spectroscopy to analyse the metabolomic changes in vitro of WLS available for purchase in Australia. Ten WLS were selected, nine WLS caused significant toxicity in HepG2 human liver cells, and of these, six met the criteria for ^1^H NMR analysis, which was based on a 25–50% reduction in cell viability. **Results**: All 10 WLS caused a significant reduction in viability of Caco-2 human intestinal cells, with seven selected for metabolic profiling. Orthogonal partial least squares discriminant analysis (O-PLS-DA) of ^1^H NMR spectral data was used to characterise the metabolites that differed between the untreated and treated cells and the fold changes of the metabolites were determined. The results showed alterations to key metabolites such as amino acids, glucose, carboxylic acids, and amines in all treatment groups compared to untreated controls across both cell lines. **Conclusions**: Collectively, these biochemical changes represent disturbances to intracellular proteins, energy metabolism, and membrane lipids suggestive of oxidative stress. This study highlights the need for further investigations into the actions of these WLS in vivo, and, as these products were regulated by the Therapeutic Goods Administration (TGA) at the time of purchase, this study suggests improved pre-market screening to ensure consumer health is protected.

## 1. Introduction

Obesity is a global health concern that is characterised by the excessive accumulation of adipose tissue and is associated with significant morbidity [1]. In 2022, 16% of adults were obese, with the worldwide prevalence of obesity doubling between 1990 and 2022 [2]. It is expected that by 2035, 51% of the global population will be overweight or obese, with an estimated $4.32 trillion AUD annual healthcare burden [3]. The prevalence of individuals who are overweight or obese is thought to be driving growth in the weight loss supplement (WLS) industry due to challenges associated with traditional forms of weight loss, such as maintaining a calorie deficit and regular exercise, as well as difficulties in accessing appropriate conventional treatments [4].

Complementary and alternative medicine (CAM) is an umbrella term for herbal medicines predominantly available for purchase off the shelf from health food stores, pharmacies, supermarkets, and online [5]. Types of CAM include traditional medicines, vitamins and minerals, homeopathy, and aromatherapy. These medicines are generally perceived to be safe due to the natural origins of their ingredients, and they typically do not undergo the stringent regulatory processes required for prescription medicines [6,7]. The use of CAM is widespread in Australia, with industry revenue reaching AUD 5.5 billion in 2022 [8]. Herbal and dietary supplements (HDS) are an increasingly popular group of CAM, with approximately 48% of the Australian population using these products regularly [9,10]. Of the HDSs, herbal weight loss supplements (WLS) are one of the fastest growing supplement categories, contributing AUD 440 million to the total CAM revenue for 2022 [8].

Despite the increasing popularity of WLS and their perceived safety, these products have been implicated in a growing number of adverse events [11]. In recent years, there has been growing evidence for the implication of HDS in cases of herb-induced liver injury (HILI), a subgroup of drug-induced liver injury (DILI) attributed to herbal products [12,13,14,15]. HILI is a liver disorder resulting from the ingestion of herbal supplements, which could be single or multi-ingredient, that leads to liver abnormalities and can subsequently lead to liver failure. Clinical presentations include fatigue, nausea, abdominal discomfort, jaundice, dark urine, and pale stools [12]. Many of the HDS commonly found to be associated with HILI have been supplements aimed at weight loss, such as green tea extract, Hydroxycut, Herbalife, and *Garcinia gummi-gutta* (L.) N.Robson (*Clusiaceae*) (also known as *G. cambogia*) [16,17,18]. The condition presents a clinical challenge and the use of diagnostic algorithms such as Roussel Uclaf Causality Assessment Method (RUCAM) is widely recommended. It is a validated method specifically designed for the determination of liver injury. However, often diagnosis of HILI is typically a process of elimination, and most cases resolve upon discontinuation of the causative HDS, nevertheless, there have been increasing incidences of progression to liver failure requiring transplantation for patient survival [11,12,13,19]. Indeed, a study by Teschke et al. demonstrated that in 80 publications up to 2020, a total of 12,068 HILI cases were identified worldwide [20]. Though the link between HDS and liver damage is clear, little is currently understood about the toxicological mechanisms involved and the factors that make certain individuals more susceptible than others.

The purpose of this study was to examine the safety of 10 WLSs currently available on the market and to identify any mechanism(s) of toxicity by analysing the biochemical changes in response to these supplements. We aimed to determine those WLSs that caused toxicity in vitro and used ^1^H nuclear magnetic resonance (NMR) spectroscopy to examine the metabolomic changes induced by the WLS in human hepatic and intestinal cells. NMR has been utilised in previous studies to detect adulterants in herbal weight loss supplements [21,22] and to monitor the metabolomic changes induced by herbal medicines [23]. There has also been a study that monitored the anti-obesity mechanism of an oriental herb *G. pentaphyllum* in obese mice through untargeted metabolomics [24]. However, to our knowledge this is the first study to monitor the metabolomic changes induced in vitro by herbal weight loss supplements available for purchase in Australia. This study is novel and the metabolomic changes observed may give insight into early identification of HILI before clinical symptoms, as well as mechanistic understanding.

## 2. Methods

### 2.1. Toxicological Screening

#### 2.1.1. WLS Preparations

Given that there are hundreds of WLS available on the market, a list of those available for purchase from Australian pharmacies, health food stores, and supplement/vitamin outlets was compiled, and 10 representative products were randomly selected for toxicological screening. At the time of purchase, seven WLS were listed on the Australian Register of Therapeutic Goods (ARTG), which is a legal requirement for the sale of these products in Australia. All supplements were crushed to a fine consistency, and serum-free DMEM containing 10% DMSO was added to create a 30 mg/mL stock solution for each. Stock solutions were diluted with serum-free DMEM to obtain final treatment concentrations of 0.1, 0.3, 1.0, and 3.0 mg/mL. A vehicle control composed of serum-free DMEM containing 1% DMSO was also prepared to match the final DMSO concentration in the treatments. The concentrations used in this study were selected based on previous research findings [25]. The WLSs will be referred to by the codes outlined in Table 1.

#### 2.1.2. Cell Culture

Human liver (HepG2) and colon (Caco-2) carcinoma cells were selected for toxicological screening due to the gastrointestinal tract being the organ system most commonly involved in adverse events relating to WLS usage. Both cell lines were obtained from the European Collection of Authenticated Cell Cultures (Salisbury, United Kingdom) (HepG2 accession number: CVCL_0027, Caco-2 accession number: CVCL_0025) and cultured in 75 cm^2^ flasks in Dulbecco’s Modified Eagle Medium (DMEM), which was supplemented with 10% foetal bovine serum (FBS), 1% non-essential amino acids, and 1% penicillin-streptomycin. Cells were incubated at 37 °C and 5% carbon dioxide and passaged 1:10 every 5–7 days, with 0.5% trypsin-EDTA used to detach cells from flasks. Trypan blue was used to stain cells for counting, which was performed manually. Cell viability experiments used 96-well plates, with cells seeded at a density of 1.2 × 10^4^ cells/well in supplemented DMEM. All plated cells were incubated for 48 h to equilibrate before experiments commenced. All cell maintenance and experiments were performed under sterile conditions in a laminar flow cabinet.

#### 2.1.3. Bioactive Pre-Treatment

The majority of drug and phytochemical metabolism is catalysed by the cytochrome P450 3A (CYP3A) enzyme family, with CYP3A4 being the most common isoform. The metabolism of phytochemicals can enhance their toxicity via the production of toxic metabolites. Given that CYP3A function is depleted in HepG2 and Caco-2 cells, it was important to examine the toxicity of WLS in the presence and absence of CYP3A4 induction. As rifampicin has been demonstrated to induce CYP3A function [26], it was added to supplemented DMEM at a concentration of 2 mM for 48 h prior to WLS exposure.

#### 2.1.4. Cell Viability

Thiazolyl blue tetrazolium bromide (MTT) cytotoxicity assay was used to analyse cell viability following exposure to WLS for 48 h. Viable cells take up MTT and convert it to an insoluble blue formazan product, with colour intensity directly proportional to mitochondrial activity in living cells. Both cell lines were incubated with WLS dilutions, including control, for 48 h. At the conclusion of the exposure period, all treated media were aspirated and replaced with serum-free medium containing 0.25 mg/mL MTT. Plates were incubated for 3 h, MTT solution was aspirated and replaced with DMSO to lyse cells. A Tecan Spark Microplate Reader was subsequently used to measure absorbance at 570 nm.

#### 2.1.5. Statistical Analysis

Statistical analysis of MTT data was completed using IBM SPSS Statistics (v29.0.1.0). MTT data were first investigated using a one-way analysis of variance (ANOVA) to determine the effects of WLS compared to the control. This was followed by a Dunnett’s post hoc test to determine *p*-values at each concentration compared to the control, with a significance of *p* < 0.01 selected for all experiments. Further data analysis and generation of cell viability graphs were carried out in Microsoft Excel (v16.75.2). Graphs depict the mean at each concentration, with significance shown as * *p* < 0.01.

### 2.2. NMR Toxicological Analysis

#### 2.2.1. WLS Preparations and Cell Culture

WLS that significantly decreased cell viability were selected for further toxicological analysis using ^1^H NMR spectroscopy. Only one concentration of each WLS was chosen, based on 25–50% reduction in cell viability (+/−1%). WLSs that did not have a concentration that fell within the defined range were excluded from further analysis. All treatment groups, including vehicle controls, were prepared as outlined above, as were HepG2 and Caco-2 cells. Six-well plates were used for ^1^H-NMR spectroscopic analysis, with cells seeded in supplemented DMEM at a density of 1.0 x 10^6^ cells/well. To best reflect in vivo responses, cells were pre-treated with rifampicin for 48 h to induce CYP3A activity. Following 48 h exposure, spent medium was removed and cells washed with PBS before being harvested into methanol (MeOH). Samples were lysed using a Bertin Technologies Precellys 24 tissue lyser at 6500 rpm for 2 × 20 s and subsequently centrifuged at 16,100 rcf for 5 min, with the supernatant transferred to fresh tubes and dried for storage at −80 °C.

#### 2.2.2. ^1^H NMR Spectroscopy Data Acquisition and Processing

All ^1^H NMR spectroscopy was carried out on a 600 MHz Bruker Avance III HD spectrometer equipped with a 5 mm BBI probe, which underwent quantitative calibration prior to analysis using a previously described protocol [27]. Cell extracts were thawed and resuspended in 540 mL D_2_O and 60 mL phosphate buffer (1.5 M KH_2_PO_4_, 2 mM NaN_3_, 0.1% TSP, pH 7.4) and transferred to a 5 mm Bruker SampleJet NMR tube which was closed with caps and sealed with POM balls. All NMR experiments were completed at 300 K. For each sample, a one-dimensional (1D) ^1^H experiment (256 scans, 65,536 data points, acquisition time of 2.73 s, and a spectral width of 12,019.23 Hz/20 ppm) was completed in automation mode, amounting to a total of 32 min acquisition time per sample.

One control sample for each cell line also underwent two-dimensional (2D) NMR experiments to elucidate chemical shift assignments. The 2D experimental parameters were ^1^H-^1^H COSY and ^1^H-^1^H TOCSY, with an analysis time of 11 h 48 min and 12 h, respectively (32 scans, 8192 data points in the F1 dimension, 512 data points in the F2 dimension, spectral width of 7812.50 Hz/13 ppm in the F1 and F2 dimensions), ^1^H-^13^C HSQC with an analysis time of 25 h 33 min (160 scans, 4096 data points in the F1 dimension, 256 data points in the F2 dimension, spectral width of 9615.385 Hz in the F1 dimension and 28,677.936 Hz/16 ppm in the F2 dimension), and ^1^H-^13^C HMBC with an analysis time of 26 h 53 min (160 scans, 4096 data points in the F1 dimension, 256 data points in the F2 dimension, spectral width of 7812.50 Hz in the F1 dimension and 34,715.75 Hz/16 ppm in the F2 dimension).

All 1D data was processed in automation using Bruker TopSpin^TM^ 3.6.2 and ICON^TM^ NMR to achieve phasing and baseline correction. All 2D data was referenced to TSP and phase-corrected manually in TopSpin.

#### 2.2.3. NMR Data Modelling

The statistical programming language R (v4.1.3) and the metabom8 package (v1.0.0) were used for all data analysis (https://tkimhofer.github.io/metabom8 (accessed on 7 September 2023)). To prepare spectral datasets for each WLS group for multivariate modelling, spectral regions corresponding to residual water resonance signal (4.60–4.85 ppm), methanol resonance signal (3.27–3.35 ppm), and noise (<0.5 and >9.5 ppm) were excluded from analyses, and the baseline was corrected using an asymmetric least squares routine for each spectrum. Spectra were normalised using a probabilistic quotient normalisation (PQN) method with the median spectrum as the reference, and data were mean-centred and scaled to unit variance prior to multivariate modelling [28]. First, principal component analysis (PCA) was performed to determine key sources of structural variation within each dataset. To identify metabolites primarily responsible for changes observed in WLS-treated cells, a previously constructed orthogonal projection to latent structure discriminant analysis (OPLS-DA) model was used [29]. This was performed for 1D spectral datasets for each WLS group separately, with the optimal number of orthogonal components selected using the area under the receiver operator curve (AUROC) calculated from predictive component scores.

^1^H and ^13^C chemical shifts for metabolites were assigned using a combination of 2D ^1^H-^1^H COSY, ^1^H-^1^H TOCSY, and ^1^H-^13^C HSQC NMR spectra with Chenomx NMR suite (v10) and HMDB (https://hmdb.ca (accessed on 4 August 2023)) and values previously reported in the literature [30,31,32].

To determine metabolite fold changes, integral regions of the metabolites in the spectrum were cut and compared between the control untreated HepG2 and Caco-2 cells and the treated cells. Fold changes above 1.0 signify an increase in the metabolite upon treatment, while fold changes below 1.0 indicate a reduction in the metabolite upon treatment. Statistical analysis of fold changes was completed using IBM SPSS Statistics (v29.0.1.0), with significance shown as * *p* < 0.05 and ** *p* < 0.01.

## 3. Results

### 3.1. Toxicological Screening

**Figure 1:**
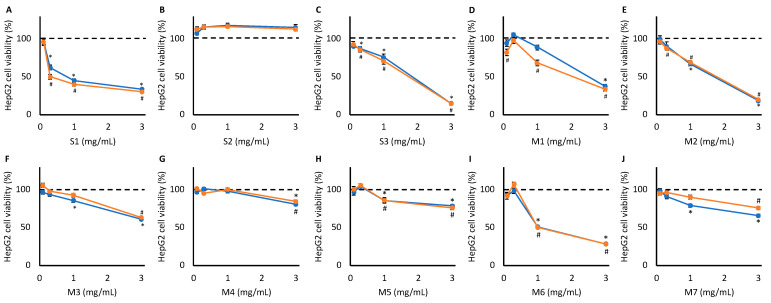
HepG2 cell viability following treatment with increasing concentrations of S1 (**A**), S2 (**B**), S3 (**C**), M1 (**D**), M2 (**E**), M3 (**F**), M4 (**G**), M5 (**H**), M6 (**I**), and M7 (**J**) for 48 h, as determined via MTT cytotoxicity assay (n = 15, *p* < 0.01). Orange: Cells were pre-treated with rifampicin to induce CYP450 activity (^#^ indicates the concentrations that cell viability is significantly different to untreated cells, *p* < 0.01). Blue: Cells did not undergo pre-treatment (* indicates the concentrations that cell viability is significantly different to untreated cells, *p* < 0.01).

In HepG2 cells, S1, S3, M1, M2, M3, M4, M5, M6, and M7 caused significant toxicity at one or more concentrations, and toxicity was generally dose-dependent (*p* < 0.01) (Figure 1). Of these, 0.3 mg S1/mL, 1.0 mg S3/mL, 1.0 mg M1/mL, 1.0 mg M2/mL, 3.0 mg M3/mL, and 1.0 mg M6/mL were the six supplement–concentration pairings that reduced HepG2 cell viability by 25–50% (+/−1%) in the rifampicin pre-treatment group and were therefore selected for ^1^H NMR spectroscopy. Although significant toxicity was observed in response to M4, M5, and M7 (*p* < 0.01), none of the concentrations tested reduced cell viability within the 25–50% window and therefore did not meet the criteria for NMR analysis.

**Figure 2:**
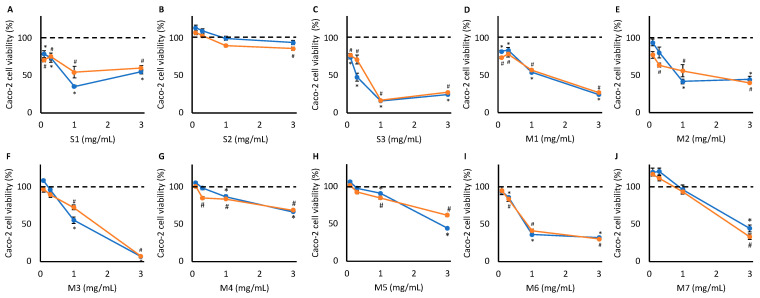
Caco-2 cell viability following treatment with increasing concentrations of S1 (**A**), S2 (**B**), S3 (**C**), M1 (**D**), M2 (**E**), M3 (**F**), M4 (**G**), M5 (**H**), M6 (**I**), and M7 (**J**) for 48 h, as determined via MTT cytotoxicity assay (n = 15, *p* < 0.01). Orange: Cells were pre-treated with rifampicin to induce CYP450 activity (^#^ indicates the concentrations that cell viability is significantly different to untreated cells, *p* < 0.01). Blue: Cells did not undergo pre-treatment (* indicates the concentrations that cell viability is significantly different to untreated cells, *p* < 0.01).

All 10 WLS tested caused significant reductions in Caco-2 cell viability at one or more concentrations, with toxicity appearing to be dose-dependent (*p* < 0.01) (Figure 2). Seven supplement–concentration pairings were selected for ^1^H NMR spectroscopy: 0.3 mg S1/mL, 0.3 mg S3/mL, 1.0 mg M1/mL, 0.3 mg M2/mL, 1.0 mg M3/mL, 3.0 mg M4/mL, and 3.0 mg M5/mL. S2, M6, and M7 did not meet the predefined criteria for NMR, and therefore were not included despite causing significant toxicity (*p* < 0.01).

A summary of the WLS concentrations selected for ^1^H NMR spectroscopy and their corresponding reductions in cell viability observed in the MTT assays can be found in Table 2.

### 3.2. NMR Analysis of In Vitro Toxicity

**Figure 3:**
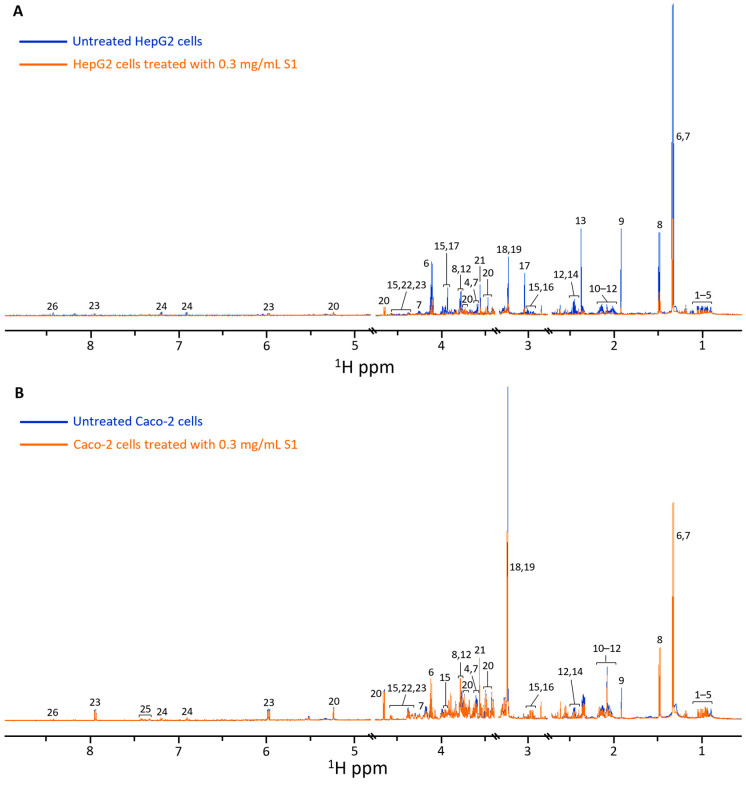
^1^H NMR spectra of (**A**) HepG2 and (**B**) Caco-2 cell extracts, comparing untreated (blue) and S1 (orange) groups. (1) 3-methyl-2-oxovalerate, (2) 2-oxoisocaproate, (3) leucine, (4) valine, (5) isoleucine, (6) lactate, (7) threonine, (8) alanine, (9) acetate, (10) proline, (11) glutamate, (12) glutamine, (13) pyruvate, (14) succinate, (15) glutathione, (16) lysine, (17) creatine, (18) phosphocholine, (19) glycerophosphocholine, (20) glucose, (21) glycine, (22) NAD^+^, (23) UDP-glucose, (24) tyrosine, (25) phenylalanine, (26) formate.

^1^H NMR spectra of S1-treated and untreated HepG2 and Caco-2 cell extracts are shown in Figure 3. Acetate, alanine, creatine, glutathione, glycine, lactate, NAD^+^, phosphocholine, pyruvate, threonine, tyrosine, and UDP-glucose were among 33 key metabolites identified in ^1^H NMR spectra of HepG2 (Figure 3A) and Caco-2 cells (Figure 3B). Though the same metabolites were identified in both cell lines, there were differences in their baseline levels in untreated HepG2 compared to Caco-2 cells.

**Figure 4:**
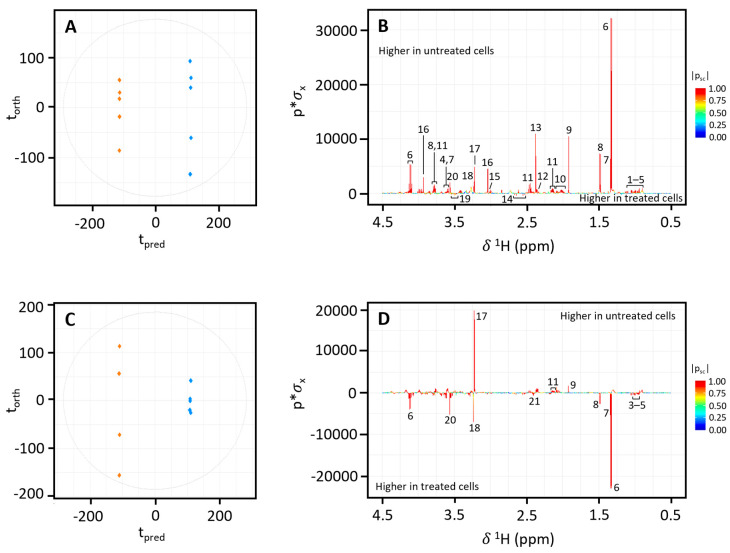
OPLS-DA score plots (CV-AUROC = 1.0) and corresponding coefficient loading plots derived from ^1^H NMR spectra of HepG2 (**A**,**B**) and Caco-2 cells (**C**,**D**) in untreated (blue) and S1 (orange) treatment groups. (1) 3-methyl-2-oxovalerate, (2) 2-oxoisocaproate, (3) leucine, (4) valine, (5) isoleucine, (6) lactate, (7) threonine, (8) alanine, (9) acetate, (10) proline, (11) glutamine, (12) glutamate, (13) pyruvate, (14) citrate, (15) lysine, (16) creatine, (17) phosphocholine, (18) glycerophosphocholine, (19) glucose, (20) glycine, (21) succinate.

OPLS-DA analysis of the NMR spectra revealed a clear difference in the metabolite profile of the HepG2 cells from each treatment group (S1, S3, M1, M2, M3, and M6) with untreated controls (Figure 4A, Supplementary Figures S1–S5). The R2X values for each model were 0.28 (S1, Figure 4A), 0.21 (S3, Supplementary Figure S1A), 0.23 (M1, Supplementary Figure S2A), 0.20 (M2, Supplementary Figure S3A), 0.22 (M3, Supplementary Figure S4A), and 0.29 (M6, Supplementary Figure S5A). AUROC and CV-AUROC were 1.0 for all six models, indicating goodness of fit and good predictability. Corresponding OPLS-DA coefficient loading plots demonstrate key metabolites contributing significantly to the observed variation between treated and untreated HepG2 cells (Figure 4B, Supplementary Figures S1B–S5B).

OPLS-DA comparing treated Caco-2 cells (S1, S3, M1, M2, M3, M4, and M5) with untreated controls demonstrated distinct differences in metabolite profiles (Figure 4C, Supplementary Figures S1–S4, S6 and S7). The R2X values for each model were 0.27 (S1, Figure 4C), 0.16 (S3, Supplementary Figure S1C), 0.24 (M1, Supplementary Figure S2C), 0.20 (M2, Supplementary Figure S3C), 0.20 (M3, Supplementary Figure S4C), 0.27 (M4, Supplementary Figure S6A), and 0.23 (M5, Supplementary Figure S7A), and AUROC and CV-AUROC were 1.0 for all seven models.

A complete overview of metabolite fold changes induced by the WLS and corresponding ^1^H and ^13^C chemical shifts in HepG2 and Caco-2 cells are outlined in Table 3 and Table 4, respectively. A significant reduction in creatine, lactate, methylmalonate, pyruvate, trimethylamine, and 2-oxoisocaproate was observed in treated HepG2 cells compared to untreated controls for all WLS (Table 3). Amino acids such as alanine, glycine, isoleucine, leucine, and valine were also present in concentrations significantly different to untreated HepG2 cells; however, whether these were increased or decreased depended upon the treatment group. The Caco-2 cells showed a more varied response in relation to WLS treatment. Glutamine levels reduced with all WLS, five of which were significantly different from the untreated cells. Significant changes in alanine, glutathione, glycine, isoleucine, leucine, and succinate were evident in five or more treatment groups compared to untreated controls (Table 4); however, these included increases and decreases in the small molecule dependent on the WLS.

## 4. Discussion

This study has highlighted significant in vitro metabolomic changes with WLS currently available for purchase on the Australian market. Nine of ten WLS examined caused significant cytotoxicity in HepG2 cells, and all ten caused significant decreases in Caco-2 cell viability. Of these, six met the criteria for ^1^H NMR analysis in HepG2 and seven in Caco-2 cells. Metabolic profiling with ^1^H NMR revealed significant changes in metabolites in each cell line, primarily involving amino acids, glucose, carboxylic acids, amines, and glutathione. Changes in metabolites such as these have previously been described in the context of oxidative stress [33].

Oxidative stress occurs when reactive oxygen species (ROS) are in excess due to an imbalance between the production and detoxification of free radicals and pro-oxidants within cells, commonly implicated in cytotoxicity [34,35]. Normally, ROS are produced as by-products of key metabolic processes such as aerobic respiration and CYP450 enzymatic reactions, and these are rapidly neutralised by endogenous antioxidants (e.g., glutathione, certain amino acids) [34,36,37]. When there is an overproduction of ROS or deficiency in cellular antioxidant mechanisms, the reduced capacity to neutralise free radicals leads to the oxidation of important macromolecules such as proteins and lipids [35,37]. A prolonged pro-oxidant state within cells leads to damage beyond repair, ultimately resulting in cell death.

Glutathione is the primary intracellular antioxidant and acts both directly in its reduced form and indirectly as a cofactor for antioxidant enzymes to protect cells against ROS [36]. All WLS induced changes in glutathione in HepG2 cells, and five of seven WLS tested did so in Caco-2 cells. In the HepG2 cells, glutathione levels increased with M1 (fc = 1.46) and M2 (1.22), and decreased with S1 (fc = 0.93), S3 (fc = 0.54), M3 (fc = 0.59), and M6 (fc = 0.84), with the changes induced by S3, M1, and M3 being significant. In the Caco-2 cells, S1 did not significantly change the glutathione level, while S3 (fc = 0.55), M1 (fc = 0.66), M2 (fc = 0.91, not significant), M3 (fc = 0.55), and M5 (fc = 0.64) significantly reduced it.

M1 and M2 in HepG2 cells yielded an increase in glutathione relative to untreated cells, likely representing increased expression in response to a pro-oxidant intracellular state [36,38]. Indeed, some studies have reported increased glutathione under oxidative stress conditions due to an increased rate of hepatic glutathione synthesis [30,39]. In most cases, glutathione levels decreased in response to WLS treatment, which may reflect the depletion of this metabolite as endogenous antioxidant mechanisms become overwhelmed, and is indicative of hepatoxicity [40,41]. Pyroglutamate, an intermediate in the g-glutamyl cycle in which glutathione is synthesised and metabolised, was significantly decreased in HepG2 cells treated with S1 (fc = 0.58), M2 (fc = 0.81), and M6 (fc = 0.59), significantly increased in Caco-2 cells for S1 (fc = 1.24), M2 (fc = 1.15) and M3 (fc = 1.21), and significantly decreased for M1 (fc = 0.86). These changes in pyroglutamate likely reflect an increase in cellular demand for glutathione [42,43]. It should be noted that different supplements result in a different response in glutathione and pyroglutamate concentrations. For example, HepG2 treated with S1 had a reduction in both glutathione and pyroglutamate while HepG2 treated with M2 had an increase in glutathione but a reduction in pyroglutamate, potentially caused by a depletion of the precursors required to produce pyroglutamate. Indeed, it was recently demonstrated that treatment of HepG2 cells with H_2_O_2_ produced a significant increase in both glutathione and pyroglutamate [30]. Further work is required to fully elucidate the mechanisms.

Many amino acids were affected by WLS treatment, including a reduction in glutamine for all WLSs, and significant differences in glycine and glutamate, which are amino acid precursors for glutathione [44,45]. Changes in these amino acids may reflect their mobilisation and consumption in response to increased need for glutathione due to oxidative stress [46,47,48]. Additionally, several amino acids such as lysine, serine, threonine, and tyrosine have been shown to have antioxidant properties [49,50,51]. All treatment groups in both cell lines had changes in at least one of these amino acids, potentially reflecting further activation of intracellular antioxidant mechanisms.

Thirteen proteinogenic amino acids were altered in treated HepG2 cells and fourteen in Caco-2 cells. Amino acids such as glycine, leucine, and valine accumulated in some treatment groups, which is suggestive of protein degradation, a common consequence of oxidative stress [34,52]. Many amino acids were decreased in treated cells, and this is thought to be in response to increased energy demands within cells [53]. Several of these were glucogenic amino acids, such as alanine, aspartate, glycine, and serine; therefore, depletion of these could be due to their use in gluconeogenesis to produce glucose [54]. Additionally, several of the amino acids affected can be converted into TCA cycle intermediates in response to a demand for alternative energy sources [53]. Examples include the conversion of glutamine and proline into α-ketoglutarate, and valine and isoleucine into succinyl CoA.

Changes in glucose and carboxylic acids in this study strengthen the theory that all tested WLSs caused disrupted energy metabolism in both cell lines. Glucose levels were significantly altered in two HepG2 (M1, fc = 1.22; M6 fc = 1.56) and three Caco-2 (S1, fc = 0.90; M1, fc = 0.73 and M5, fc = 0.69) treatment groups. Oxidative stress can be associated with the oxidation and subsequent inhibition of glycolytic enzymes such as glyceraldehyde-3-phosphate dehydrogenase, resulting in a decrease in glycolysis [55]. Therefore, the accumulation of glucose observed in HepG2 cells likely reflects a disruption in glycolysis, a theory which is supported by corresponding decreases in pyruvate and lactate observed in this cell line. On the other hand, treated Caco-2 cells demonstrated decreased glucose and elevated pyruvate and lactate, indicating increased glycolytic function and anaerobic respiration in these cells. This could be in response to increased energy demands in the presence of disrupted oxidative phosphorylation.

Aerobic respiration appeared to be impacted in both cell lines, with alterations observed in TCA cycle intermediates such as citrate, succinate, and fumarate. Caco-2 cells treated with S1 (fc = 1.50) and HepG2 treatment groups S1 (fc = 2.28), S3 (fc = 1.40), M1 (fc = 2.31), and M6 (fc = 1.66) showed significant increases in intracellular citrate, which is the first intermediate in the TCA cycle. This accumulation of citrate could be due to oxidative damage to TCA cycle-related enzymes, particularly aconitase [56]. Furthermore, it is well documented that high levels of citrate inhibit phosphofructokinase; therefore, this could be contributing to the reduced glycolytic function observed in treated HepG2 cells [57]. Citrate has also been shown to indirectly enhance oxidative stress in HepG2 cells by promoting iron free radical formation, thought to be a result of iron-citrate complexes being more prone to hydroxyl radical formation [58,59]. Caco-2 cells also demonstrated decreased succinate levels in S1 (fc = 0.72), S3 (fc = 0.82) and M1 (fc = 0.53) treatment groups, increased fumarate in S1-treated cells, and decreased fumarate in cells exposed to M2, increasing the likelihood that the TCA cycle has been impacted in this cell line. This in turn further supports the theory that treated Caco-2 cells had increased reliance on anaerobic mechanisms for energy generation.

Other metabolites involved in energy metabolism that were impacted in this study include acetate, formate, and methylmalonate. Acetate levels were decreased in all HepG2 and three Caco-2 treatment groups and increased in Caco-2 cells treated with S3, M1, M3, and M5. Acetate is a metabolite that can be produced from pyruvate and is central to both the anabolic and catabolic metabolism of carbohydrates and fats, particularly in both the synthesis and degradation of acetyl-CoA [60]. Changes were also observed in formate, where it was reduced in the HepG2 cells with the treatment groups and increased in the Caco-2 cells. Formate can be produced in a variety of ways, for example, as a byproduct of acetate synthesis or serine metabolism, and in anaerobic systems can be produced through the reduction of carbon dioxide. This metabolite has inhibitory effects on the mitochondrial electron transport chain, potentially further disrupting aerobic respiration in these cells [61]. Methylmalonate was found to be significantly decreased in the HepG2 cells in all treatment groups and increased in four treatment groups in the Caco-2 cells. Methylmalonate is normally produced through the metabolism of propionyl-CoA, which is derived from the breakdown of amino acids such as isoleucine, methionine, threonine, and serine [62]. Elevated levels could suggest enhanced breakdown of proteins [62]. Methylmalonate can also be used to synthesise methylmalonyl-CoA via conjugation with coenzyme A, which can then be converted into succinyl-CoA and fed into the TCA cycle [63]. Therefore, decreased levels of methylmalonate likely represent increased demand for alternative fuel sources.

Alterations in lipid metabolism were also evident. A decrease in phosphocholine was seen in all HepG2-treated cells and predominantly in Caco-2 cells with only treatment M3 increasing these levels. A reduction in phosphocholine can affect the overall lipid content on the cells and impact cellular processes such as membrane structure and signalling [64]. It has been shown that choline depletion can reduce the production of phosphocholine [64]. Although choline was not measured in this study, serine, which can be a precursor to choline synthesis, was significantly reduced [65].

This study used immortalised cell lines to examine WLS toxicity; three key advantages to this approach are that 1) they bypass the requirement for animal models, creating a more ethical method for screening substances which may cause harm, 2) they are easier and more efficient to grow than primary cell lines, and 3) their genetic homogeneity yields reproducible results [66]. However, key limitations associated with immortalised cells lines include the genetic changes required for indefinite proliferation, leading to changes in cell function, and the lack of 3D arrangement with other cell types ordinarily found in vivo, resulting in different cell–cell interactions [26,67]. Other limitations of this study include limited analysis of dose–response relationships and a lack of compound-specific toxicity profiling. The majority of WLSs demonstrated a dose-dependent decline in cell viability, and while not the focus of this study, future research analysing the change in metabolite profile across different concentrations would be beneficial in further elucidating biochemical responses to supplements such as these. It would also be valuable for active compounds commonly included in WLS to undergo safety profiling, including the exploration of compound–compound interactions, to further understand the toxicity observed in this study and predict safe combinations for future inclusion in WLS.

## 5. Conclusions

This study used untargeted ^1^H NMR spectroscopy to determine changes in the metabolic profiles of HepG2 and Caco-2 cells in response to herbal WLS available on the Australian market. Metabolites that were most impacted included glucose, carboxylic acids, amino acids, phosphocholine, and glycerophosphocholine. Collectively, these changes suggest that WLS induced disrupted energy metabolism, protein degradation, and alterations in lipid metabolism. Given that WLS-induced changes occurred at relatively low concentrations, this demonstrates that individuals using these products may be at risk of adverse events such as hepatotoxicity. As seven of the WLS that caused significant toxicity in vitro were under TGA regulation at the time of purchase, this suggests that increased screening needs to be completed prior to products coming to market to ensure consumer health.

This study has highlighted both the toxicological and metabolomic effects of HWS. While this study has been completed in vitro, the findings warrant additional studies to understand the in vivo effects at the recommended dose levels. In addition, many HWS are self-prescribed, and the toxicological and metabolomic effects when they may interact with prescribed pharmaceutical drugs are not yet known, but should be fully investigated.

## Figures and Tables

**Table 1:** ARTG status of WLS included in this study and their active ingredients as listed on product labels. Code prefix S denotes a single-ingredient product and M indicates a multi-ingredient supplement.

WLS Code	Active Ingredients(s)	ARTG Status
S1	*Camellia sinensis* (L.) Kuntz (*Theaceae*) (green tea catechins (GTCs), caffeine)	Listed
S2	*G. gummi-gutta* (hydroxycitric acid (HCA))	Listed
S3	*Coffea canephora* (chlorogenic acid (CGA), caffeine)	Listed
M1	*C. sinensis* (GTCs, caffeine); *Coffea canephora* Pierre ex A.Froehner (*Rubiaceae*) (CGA, caffeine); caffeine anhydrous; *Capsicum annuum* (L.) (*Solanaceae*) (capsaicin); *Citrus aurantium* L. (*Rutaceae*) (synephrine); “Theacrine”; “Dynamine”; Higenamine HCL; *Piper nigrum* L. (*Piperaceae*) (piperine); chromium picolinate	Not listed
M2	Taurine; *Paullinia cupana* Kunth (*Sapindaceae*) (guarana)*;* tyrosine; *Theobroma cacao* L. (*Malvaceae*) (theobromine, caffeine)*;* thiamine (vitamin B_1_)*; C. sinensis* (GTCs, caffeine)*; C. aurantium* (synephrine)*; Nandina domestica* Thunb. (*Berberidaceae*); Siberian *Rhodiola rosea* L. (*Crassulaceae*)*; Tetradium* Lour. (*Rutaceae*) extract; naringen	Not listed
M3	Medium chain triglyceride (MCT) powder, decaffeinated *C. canephora* (CGA), L-carnitine, *P. cupana* (guarana), *C. annuum* (capsaicin)	Not listed
M4	*Caralluma fimbriata* Wall. (*Apocynaceae*) herb extract; *G. gummi-gutta* (HCA); *C. aurantium* (synephrine), chromium chloride hexahydrate; chromium picolinate	Listed
M5	*C. sinensis* (GTCs, caffeine)*; Fucus vesiculosus;* chromium; thiamine (vitamin B_1_); riboflavin (vitamin B_2_); nicotinamide (vitamin B_3_); pyridoxine (vitamin B_6_); cyanocobalamin (vitamin B_12_)*; Plantago afra* L. (*Plantaginaceae*)*; Garcinia quaesita* Pierre (*Clusiaceae*)*; P. cupana* (guarana)*; C. aurantium* (synephrine)*; Eleutherococcus senticosus* (Rupr. & Maxim.) Maxim. (*Araliaceae*) (Siberian ginseng)*; Coleus barbatus* (Andrews) Benth. ex G.Don (*Lamiaceae*) (forskolin)*; Ilex paraguariensis* A.St.-Hil. (*Aquifoliaceae*)*; C. canephora* (CGA, caffeine)	Listed
M6	*Moringa oleifera* Lam. (*Moringaceae*), *Bergera koenigii* L. (*Rutaceae*) (curry tree extract), *Curcuma longa* L. (Zingiberaceae) (curcumin), potassium iodide, chromic chloride hexahydrate, *C. sinensis* (GTCs, caffeine), *P. cupana* (guarana), caffeine, riboflavin (Vitamin B_2_), nicotinamide (Vitamin B_3_), calcium pantothenate, *C. annuum* (capsaicin)	Listed
M7	*C. canephora* (CGA, caffeine), *T. cacao* (theobromine, caffeine), caffeine, *C. barbatus* (forskolin)*, C. annuum* (capsaicin)	Listed

**Table 2:** Summary of WLS concentrations selected for ^1^H NMR spectroscopy and corresponding reduction in HepG2 and Caco-2 cell viability.

Weight Loss Supplement	HepG2 Cells		Caco-2 Cells	
	Concentration (mg/mL)	Reduction in Cell Viability (%)	Concentration (mg/mL)	Reduction in Cell Viability (%)
S1	0.3	49.8	0.3	24.3
S3	1.0	28.8	0.3	28.6
M1	1.0	31.6	1.0	42.7
M2	1.0	30.9	0.3	36.3
M3	3.0	36.5	1.0	27.1
M4	-	-	3.0	31.3
M5	-	-	3.0	38.0
M6	1.0	49.4	-	-

**Table 3:** Metabolite changes in HepG2 cells identified via ^1^H NMR analysis of cell extracts following treatment with six WLS (S1, S3, M1, M2, M3, and M6) for 48 h. Fold changes (fc) indicate metabolite abundance in treated HepG2 cells relative to untreated cells, with values < 1.0 signifying a decrease and values > 1.0 reflecting an increase. Statistically significant differences in metabolites in treated cells compared to untreated cells are denoted by * *p* < 0.05 and ** *p* < 0.01. Peak multiplicity singlet (s), doublet (d), double of doublets (dd), triplet (t), quartet (q), and multiplet (m).

Metabolites	δ ^1^H ppm and Multiplicity	δ ^13^C ppm	Fold Change Relative to Untreated Cells					
			S1	S3	M1	M2	M3	M6
Acetate	1.92 (s)	26.10	0.15 **	0.67 *	0.24 **	0.47 **	0.69	0.17 **
Alanine	1.49 (d), 3.78 (q)	17.20, 54.60	0.33 **	-	-	0.63 *	-	0.20 **
Citrate	2.54 (d), 2.69 (d)	48.70, 48.70	2.28 **	1.40 **	2.31 **	0.99	-	1.66 **
Creatine	3.04 (s), 3.93 (s)	40.10, 56.24	0.22 **	0.87 *	0.66 **	0.64 *	0.48 **	0.12 **
Formate	8.43 (s)	172.10	0.43 **	-	0.75 **	0.87	0.69 *	0.43 **
Glucose	3.25 (dd), 3.41 (t), 3.42 (t), 3.47 (m), 3.50 (t), 3.54 (dd), 3.72 (t), 3.73 (dd), 3.77 (dd), 3.83 (m), 3.84 (m), 3.90 (dd), 4.65 (d), 5.24 (d)	77.02, 73.50, 78.65, 63.53, 74.15, 63.32, 63.39, 96.00	1.22 *	1.02	1.05	0.90	-	1.56 **
Glutamate	2.05 (m), 2.14 (m), 2.34 (m), 2.37 (m), 3.76 (q)	29.70, 36.24, 57.57	1.24 *	1.05	1.19 **	0.98	0.92	1.57
Glutamine	2.14 (m), 2.46 (m), 3.78 (t)	29.50, 32.93, 57.10	0.15 **	0.88	0.86 *	0.76	0.71	0.14 **
Glutathione	2.16 (m), 2.19 (m), 2.54 (m), 2.57 (m), 2.93 (dd), 2.98 (dd), 3.76 (dd), 3.77 (t), 3.80 (dd), 4.57 (dd), 8.26 (m), 8.51 (s)	29.06, 34.05, 28.32, 46.35, 57.17, 59.21, 58.55	0.93	0.54 **	1.46 *	1.22	0.59 *	0.84
Glycerophosphocholine	3.23 (s), 3.69 (m), 3.92 (m), 4.32 (dd)	55.90, 68.10, 73.90, 64.40	0.65 **	1.56 **	2.00 **	1.19	2.37 *	0.38 **
Glycine	3.56 (s)	44.50	0.50 **	1.25 **	1.52 **	0.88	-	0.31 **
Isobutyrate	1.13 (d), 2.38 (m)	22.01, 39.58	0.51 **	-	0.56 **	0.75	0.66 *	0.47 **
Isoleucine	0.96 (t), 1.02 (d), 1.26 (m), 1.47 (m), 1.98 (m), 3.67 (d)	13.91, 17.37, 27.00	0.71 **	1.25 **	1.19 **	-	1.17	0.68 *
Lactate	1.33 (d), 4.11 (q)	20.20, 68.60	0.31 **	0.74 **	0.58 **	0.59 **	0.62 *	0.17 **
Leucine	0.96 (d), 0.97 (d), 1.68 (m), 1.71 (m), 1.73 (m), 3.74 (dd)	23.40, 24.30, 42.60, 56.90	0.69 **	1.34 **	1.28 **	-	1.26	0.70 *
Lysine	1.44 (m), 1.48 (m), 1.72 (m), 1.88 (m), 1.92 (m), 3.02 (t), 3.77 (t)	24.04, 29.15, 42.12, 57.45	0.52 **	0.94	0.72 *	0.84	-	0.57 **
Methylamine	2.61 (s)	28.30	0.48	0.81	0.63 *	0.59	0.62	0.39 **
Methylmalonate	1.22 (d), 3.17 (q)	17.91	0.45 **	0.70 **	0.58 **	0.56 **	0.56 **	0.38 **
NAD+	4.22 (m), 4.24 (m), 4.26 (m), 4.37 (m), 4.38 (m), 4.43 (m), 4.49 (t), 4.51 (dd), 4.54 (m), 4.77 (t), 6.04 (d), 6.10 (d), 8.18 (s), 8.20 (m), 8.43 (s), 8.83 (m), 9.15 (d), 9.35 (s))	68.13, 73.47, 80.47, 102.73, 131.34	0.67 **	-	0.85 **	0.94	0.82 *	0.62 **
Phenylalanine	3.13 (dd), 3.29 (m), 3.98 (m), 7.34 (m), 7.38 (m), 7.43 (m)	38.90, 38.90, 58.90, 132.20, 129.80, 131.90	-	1.13 *	1.15 **	1.51 *	1.21	-
Phosphocholine	3.22 (s), 3.60 (m), 4.15 (m)	56.52, 68.90, 60.61	0.24 **	0.86	0.56 **	0.67	0.63 *	0.19 **
Proline	1.99 (m), 2.03 (m), 2.07 (m), 2.37 (m), 3.34 (m), 3.42 (m), 4.13 (dd)	24.84, 30.13, 47.42, 62.29, 175.27	0.48 **	1.46 *	1.27 **	0.74 *	1.09	0.48 **
Pyroglutamate	2.03 (m), 2.39 (m), 2.42 (m), 2.50 (m), 4.17 (dd)	32.28, 27.98, 60.97	0.58 **	-	-	0.81 *	-	0.59 **
Pyruvate	2.38 (s)	29.50	0.08 **	0.50 *	0.19 **	0.46 *	0.40 *	0.05 **
Serine	3.85 (dd), 3.96 (d), 3.99 (d), 4.01 (d)	57.40, 61.31, 173.37	0.35 **	-	0.74 **	0.64 *	0.77	0.24 **
Threonine	1.34 (d), 3.59 (d), 4.26 (m)	22.30, 63.46	0.37 **	-	0.89	0.78	0.79	0.31 **
Trimethylamine	2.85 (s)	47.55	0.46 **	0.70 **	0.60 **	0.57 *	0.58 *	0.36 **
Tyrosine	3.06 (dd), 3.20 (dd), 3.97 (dd), 6.91 (m), 7.20 (m)	37.90, 59.40, 118.80, 133.40	0.62 **	-	-	0.83	-	0.56 **
UDP-glucose	3.47 (t), 3.55 (m), 3.77 (t), 3.79 (dd), 3.86 (dd), 3.89 (m), 4.20 (m), 4.24 (m), 4.29 (m), 4.36 (m), 4.38 (m), 5.60 (dd), 5.98 (d), 5.99 (d), 7.96 (d)	60.08, 64.69, 68.94, 71.33, 73.52, 82.96, 88.19, 95.31, 102.36, 141.18, 151.26, 164.21	0.76 **	1.65 **	1.26 **	1.09	1.43	0.61 **
Valine	0.99 (d), 1.05 (d), 2.28 (m), 3.61 (d)	19.41, 20.75, 31.89	0.57 **	1.20 **	1.07	-	-	0.54 **
2-oxoisocaproate	0.94 (d), 2.08 (m), 2.60 (d)	22.40, 22.40, 24.20, 47.90, 161.57, 194.07	0.25 **	0.80 **	0.53 **	0.57 *	0.52 **	0.17 **
3-methyl-2-oxovalerate	0.90 (t), 1.10 (d), 1.45 (m), 1.69 (m), 2.93 (m)	11.20, 14.40, 24.70, 43.40, 162.25, 195.00	0.47 **	0.79 **	0.57 **	0.78	0.65 **	0.45 **

**Table 4:** Metabolite changes in Caco-2 cells identified via ^1^H NMR analysis of cell extracts following treatment with six WLS (S1, S3, M1, M2, M3, M4, and M5) for 48 h. Fold changes < 1.0 indicate a decrease in metabolite abundance relative to untreated Caco-2 cells, while values > 1.0 indicate an increase. Statistically significant differences in metabolites in treated cells compared to untreated is cells denoted by * *p* < 0.05 and ** *p* < 0.01. Peak multiplicity singlet (s), doublet (d), double of doublets (dd), triplet (t), quartet (q), and multiplet (m).

Metabolites	δ ^1^H ppm and Multiplicity	δ ^13^C ppm	Fold Change Relative to Untreated Cells						
			S1	S3	M1	M2	M3	M4	M5
Acetate	1.92 (s)	26.10	0.65 **	1.08	1.14	0.71 **	1.36 **	0.83 *	1.21
Alanine	1.49 (d), 3.78 (q)	17.21, 54.60	1.28 **	-	0.76 **	0.80 **	1.27 **	1.58 **	1.10
Asparagine	2.88 (dd), 2.98 (dd), 3.99 (t)	35.70, 52.40, 174.10, 175.30	0.99	-	0.82	0.90	-	-	0.82
Aspartate	2.68 (dd), 2.80 (dd), 3.90 (t)	39.30, 55.09	1.24 **	-	0.65 **	0.78 **	-	-	0.69 **
Citrate	2.54 (d), 2.65 (d)	48.70, 48.70	1.50 **	-	1.43	1.08	-	0.83 *	-
Formate	8.43 (s)	172.10	-	-	1.17 **	1.09	-	1.14 **	1.16 **
Fumarate	6.52 (s)	132.45, 166.73	1.38	-	-	0.75 *	-	-	-
Glucose	3.25 (dd), 3.41 (t), 3.42 (t), 3.47 (m), 3.50 (t), 3.54 (dd), 3.72 (t), 3.73 (dd), 3.77 (dd), 3.83 (m), 3.84 (m), 3.90 (dd), 4.65 (d), 5.24 (d)	77.02, 73.50, 78.65, 63.53, 74.15, 63.32, 63.39, 96.00	0.90 **	0.93	0.73 **	1.09	0.97	1.01	0.69 **
Glutamate	2.05 (m), 2.14 (m), 2.34 (m), 2.37 (m), 3.76 (q)	29.70, 36.24, 57.57	0.73 **	0.90 **	0.58 **	1.02	0.88 **	0.52 **	0.60 **
Glutamine	2.14 (m), 2.46 (m), 3.78 (t)	29.51, 32.94, 57.10	0.67 **	0.76 **	0.40 **	0.86 **	0.95	0.52 **	0.46 **
Glutathione	2.16 (m), 2.19 (m), 2.54 (m), 2.57 (m), 2.93 (dd), 2.98 (dd), 3.76 (dd), 3.77 (m), 3.80 (dd), 4.57 (dd), 8.27 (m), 8.51 (s)	29.06, 34.05, 28.32, 46.35, 57.17, 59.21, 58.55	1.04	0.55 **	0.66 **	0.91 *	0.55 **	-	0.64 **
Glycerophosphocholine	3.23 (s), 3.69 (m), 3.92 (m), 4.32 (dd)	55.90, 68.10, 73.90, 64.40	1.29 **	1.04	1.21	1.27 *	1.25 **	0.45 **	1.07
Glycine	3.56 (s)	44.50	2.13 **	0.78 **	0.86	1.18	0.81	0.92	0.62 **
Isoleucine	0.96 (t), 1.02 (d), 1.26 (m), 1.47 (m), 1.98 (m), 3.67 (d)	13.91, 17.37, 27.00	1.58 **	-	0.83 **	1.25 **	1.20 **	1.61 **	0.85 *
Lactate	1.33 (d), 4.11 (q)	20.20, 68.60	2.26 **	1.14	1.85 **	0.95	1.54	3.21 **	2.04 **
Leucine	0.96 (d), 0.97 (d), 1.68 (m), 1.71 (m), 1.73 (m), 3.74 (dd)	23.40, 24.30, 42.60, 56.90	1.40 **	-	0.68 **	1.20 **	1.14 **	1.57 **	0.71 **
Lysine	1.44 (m), 1.48 (m), 1.72 (m), 1.88 (m), 1.92 (m), 3.02 (t), 3.77 (t)	24.04, 29.15, 42.12, 57.45	-	-	-	0.81	-	-	-
Methylamine	2.61 (s)	28.30	-	-	-	-	-	-	0.74
Methylmalonate	1.22 (d), 3.17 (q)	17.91	1.28 *	-	1.33 *	-	-	1.80 **	1.34 *
Myoinositol	3.28 (t), 3.54 (dd), 3.63 (t), 4.07 (t)	75.24	2.39 **	0.89	0.70 **	1.30 **	-	-	-
NAD+	4.22 (m), 4.24 (m), 4.26 (m), 4.37 (m), 4.38 (m), 4.43 (m), 4.49 (t), 4.51 (dd), 4.54 (m), 4.77 (t), 6.04 (d), 6.10 (d), 8.18 (s), 8.20 (m), 8.43 (s), 8.83 (m), 9.15 (d), 9.35 (s)	68.13, 73.47, 80.47, 102.73, 131.34	-	-	1.12	1.15	-	1.11	-
Pantothenate	0.88 (s), 0.92 (s), 2.42 (t), 3.39 (d), 3.44 (q), 3.44 (q), 3.51 (d), 3.98 (s), 8.01 (m)	21.83, 23.15, 39.08, 71.21, 38.80, 71.34, 78.65	0.41 **	1.02	-	-	0.72 **	0.87	0.79
Phenylalanine	3.13 (dd), 3.29 (m), 3.98 (m), 7.34 (m), 7.38 (m), 7.43 (m)	38.90, 38.90, 58.90, 132.20, 129.80, 131.90	1.07	-	1.50 **	-	-	1.58 **	0.96
Phosphocholine	3.22 (s), 3.60 (m), 4.15 (m)	56.52, 68.90, 60.61	0.62 **	0.95	0.84	0.79 **	0.89	1.18 **	0.95
Pyroglutamate	2.03 (m), 2.39 (m), 2.42 (m), 2.50 (m), 4.17 (dd)	32.28, 27.98, 60.97	1.24 **	0.98	0.86 *	1.15 **	1.21 **	1.04	1.01
Pyruvate	2.38 (s)	29.50	1.10	-	1.48	0.83	1.46	1.59 **	-
Serine	3.85 (dd), 3.96 (d), 3.99 (d)	57.40, 61.31, 173.37	-	-	-	0.84 *	-	-	-
Succinate	2.41 (s)	37.30	0.72 **	0.82 *	0.53 **	-	1.08	-	-
Threonine	1.34 (d), 3.59 (d), 4.26 (m)	22.30, 63.46	0.67 **	0.95	0.83	0.79 **	0.93	-	0.95
Trimethylamine	2.85 (s)		-	-	-	-	-	-	0.71
Tyrosine	3.06 (dd), 3.20 (dd), 3.97 (dd), 6.91 (m), 7.20 (m)	37.90, 59.40, 118.80, 133.40	1.20 **	-	0.90	-	-	1.19 **	0.80 **
UDP-glucose	3.47 (t), 3.55 (m), 3.77 (t), 3.79 (dd), 3.86 (dd), 3.89 (m), 4.20 (m), 4.24 (m), 4.29 (m), 4.36 (m), 4.38 (m), 5.60 (dd), 5.98 (d), 5.99 (d), 7.96 (d)	60.08, 64.69, 68.94, 71.33, 73.52, 82.96, 88.19, 95.31, 102.36, 141.18, 151.26, 164.21	0.84 **	0.97	0.91	1.05	0.96	0.53 **	1.05
Valine	0.99 (d), 1.05 (d), 2.28 (m), 3.61 (d)	19.41, 20.75, 31.89	1.82 **	-	0.91	1.34 **	1.21 *	1.52 **	0.81 *

## Data Availability

The data that support the findings of this study are available at DOI: 10.5281/zenodo.17014908.

## Supplementary Materials

The following supporting information can be downloaded at: https://www.mdpi.com/article/10.3390/metabo15090587/s1, Figure S1: OPLS-DA scores plots (CV-AUROC = 1.0) and corresponding coefficient loadings plots derived from ^1^H-NMR spectra of HepG2 (A, B) and Caco-2 cells (C, D) in untreated (blue) and S3 (orange) treatment groups; Figure S2: OPLS-DA scores plots (CV-AUROC = 1.0) and corresponding coefficient loadings plots derived from ^1^H-NMR spectra of HepG2 (A, B) and Caco-2 cells (C, D) in untreated (blue) and M1 (orange) treatment groups; Figure S3: OPLS-DA scores plots (CV-AUROC = 1.0) and corresponding coefficient loadings plots derived from ^1^H-NMR spectra of HepG2 (A, B) and Caco-2 cells (C, D) in untreated (blue) and M2 (orange) treatment groups; Figure S4: OPLS-DA scores plots (CV-AUROC = 1.0) and corresponding coefficient loadings plots derived from ^1^H-NMR spectra of HepG2 (A, B) and Caco-2 cells (C, D) in untreated (blue) and M3 (orange) treatment groups; Figure S5: OPLS-DA scores plot (A, CV-AUROC = 1.0) and corresponding coefficient loadings plot (B) derived from ^1^H-NMR spectra of HepG2 cells treated with M6 for 48 h (orange) compared to untreated cells (blue); Figure S6: OPLS-DA scores plot (A, CV-AUROC = 1.0) and corresponding coefficient loadings plot (B) derived from ^1^H-NMR spectra of Caco-2 cells treated with M4 for 48 h (orange) compared to untreated cells (blue); Figure S7: OPLS-DA scores plot (A, CV-AUROC = 1.0) and corresponding coefficient loadings plot (B) derived from ^1^H-NMR spectra of Caco-2 cells treated with M5 for 48 h (orange) compared to untreated cells (blue). 
