# Supplementary material for: Herbal Weight Loss Supplements Induce Metabolomic In Vitro Changes Indicative of Oxidative Stress
Source: Metabolites. 2025 Sep 1;15(9):587. doi: 10.3390/metabo15090587 (PMC12471570; doi:10.3390/metabo15090587)
Supplement: Supplementary file 1 [file metabolites-15-00587-s001.zip › metabolites-3813642-supplementary.pdf]

Herbal weight loss supplements induce metabolomic *in-vitro* changes, indicative of oxidative stress

Emily C. Davies[1,2], Garth L. Maker[1,2], Ian F. Musgrave[3], Samantha Lodge[1,4]

[1]Centre for Computational and Systems Medicine, Murdoch University, Perth, WA 6150, Australia

[2]Medical, Molecular and Forensic Sciences, Murdoch University, 90 South Street, Murdoch, WA 6150, Australia

[3]Adelaide Medical School, The University of Adelaide, Adelaide, SA 5005, Australia

[4]Australian National Phenome Centre, Health Futures Institute, Murdoch University, Harry Perkins Building, Perth, WA 6150, Australia

Correspondence to:
Samantha Lodge: sam.lodge@murdoch.edu.au
Garth Maker G.Maker@murdoch.edu.au

Key words: herbal weight loss supplements, ^{1}H NMR spectroscopy, HepG2 cells, Caco-2 Cell, metabolomics, oxidative stress.

[OFFICIAL]

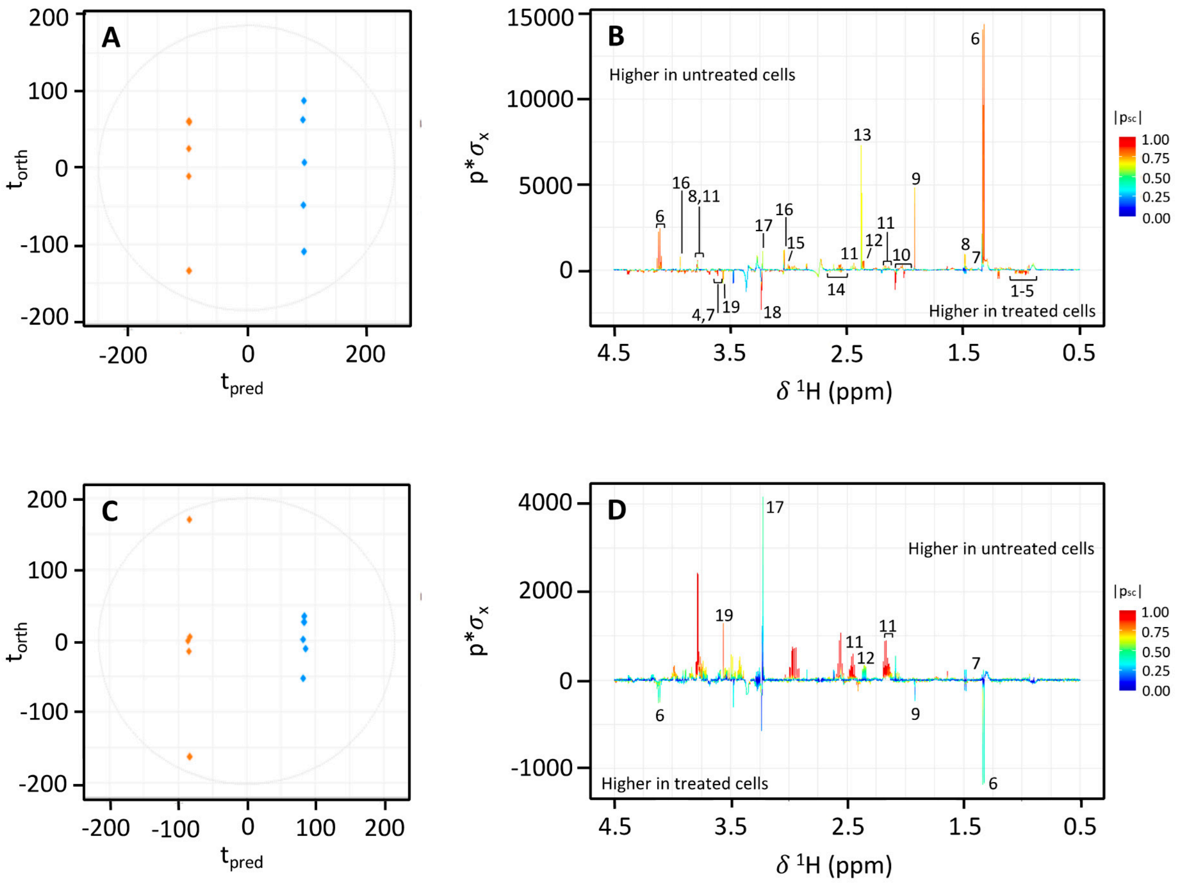

Figure S1. OPLS-DA scores plots (CV-AUROC=1.0) and corresponding coefficient loadings plots derived from ^{1}H-NMR spectra of HepG2 (A, B) and Caco-2 cells (C, D) in untreated (blue) and S3 (orange) treatment groups. (1) 3-methyl-2-oxovalerate, (2) 2-oxoisocaproate, (3) leucine, (4) valine, (5) isoleucine, (6) lactate, (7) threonine, (8) alanine, (9) acetate, (10) proline, (11) glutamine, (12) glutamate, (13) pyruvate, (14) citrate, (15) lysine, (16) creatine, (17) phosphocholine, (18) glycerophosphocholine, (19) glycine.

[OFFICIAL]

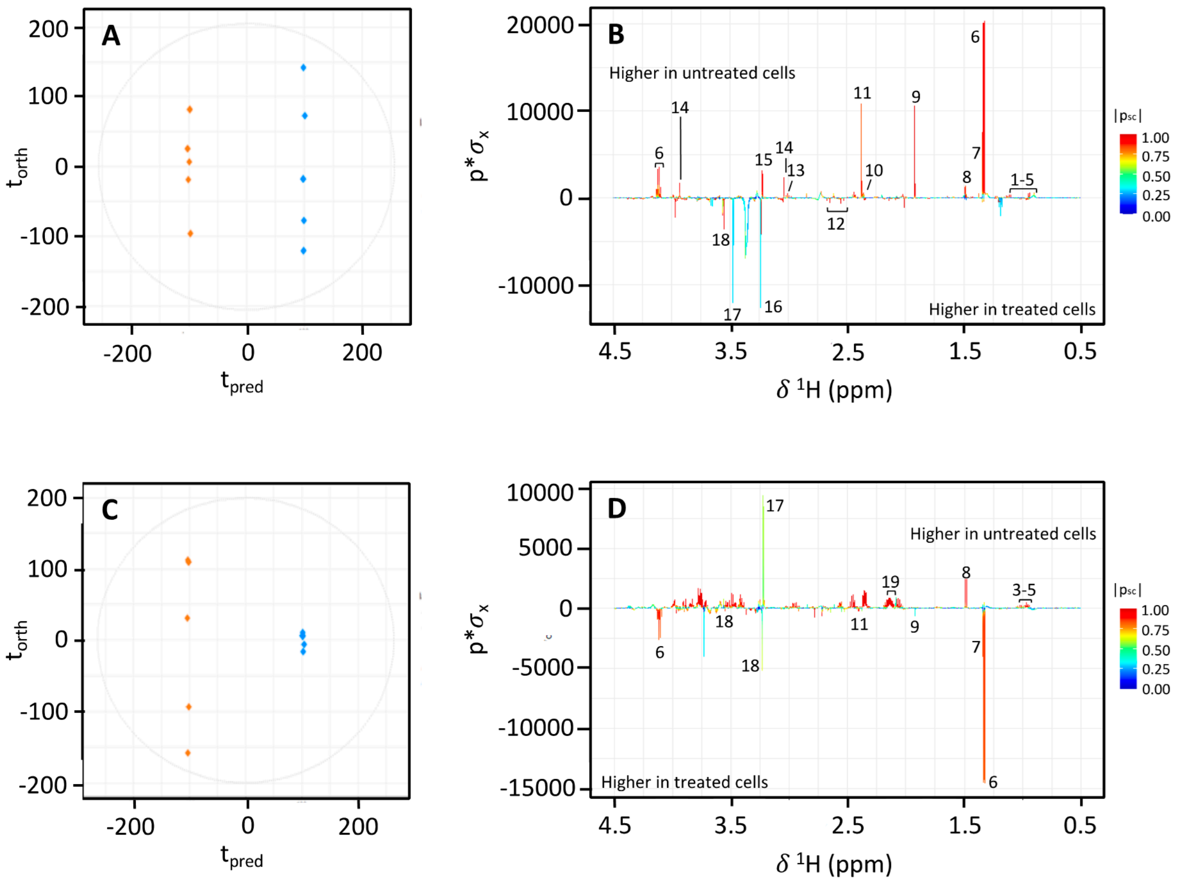

Figure S2. OPLS-DA scores plots (CV-AUROC=1.0) and corresponding coefficient loadings plots derived from ^{1}H-NMR spectra of HepG2 (A, B) and Caco-2 cells (C, D) in untreated (blue) and M1 (orange) treatment groups. (1) 3-methyl-2-oxovalerate, (2) 2-oxoisocaproate, (3) leucine, (4) valine, (5) isoleucine, (6) lactate, (7) threonine, (8) alanine, (9) acetate, (10) glutamate, (11) pyruvate, (12) citrate, (13) lysine, (14) creatine, (15) phosphocholine, (16) glycerophosphocholine, (17) glucose, (18) glycine, (19) glutamine.

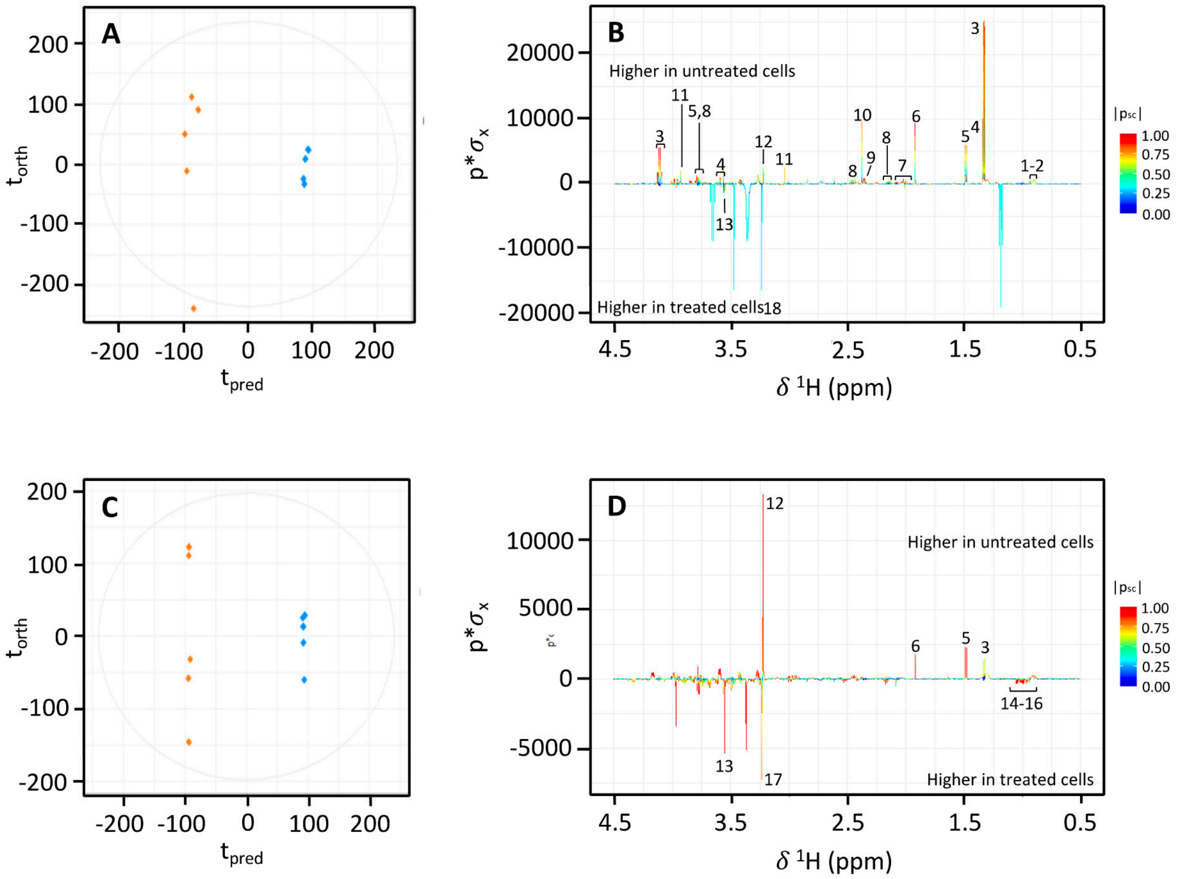

Figure S3. OPLS-DA scores plots (CV-AUROC=1.0) and corresponding coefficient loadings plots derived from ^{1}H-NMR spectra of HepG2 (A, B) and Caco-2 cells (C, D) in untreated (blue) and M2 (orange) treatment groups. (1) 3-methyl-2-oxovalerate, (2) 2-oxoisocaproate, (3) lactate, (4) threonine, (5) alanine, (6) acetate, (7) proline, (8) glutamine, (9) glutamate, (10) pyruvate, (11) creatine, (12) phosphocholine, (13) glycine, (14) leucine, (15) valine, (16) isoleucine, (17) glycerophosphocholine.

[OFFICIAL]

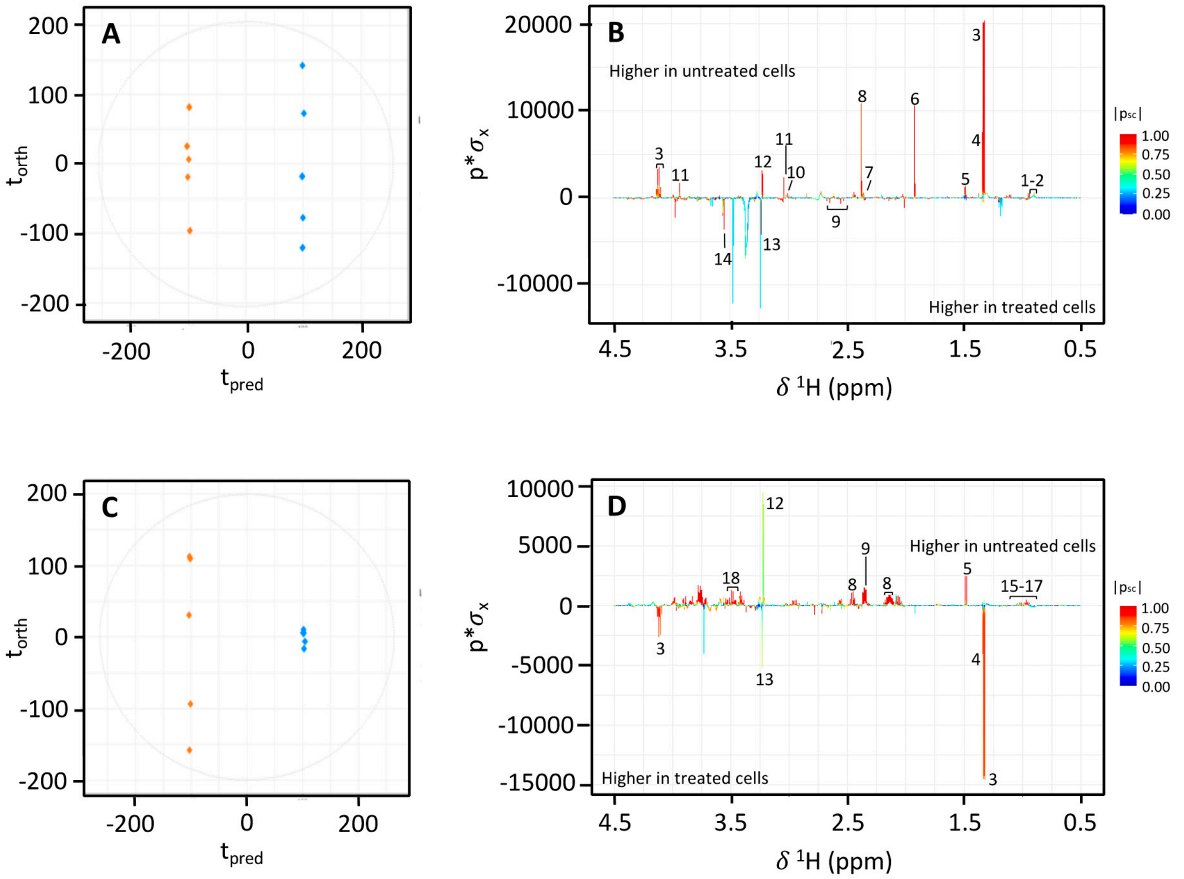

Figure S4. OPLS-DA scores plots (CV-AUROC=1.0) and corresponding coefficient loadings plots derived from ^{1}H-NMR spectra of HepG2 (A, B) and Caco-2 cells (C, D) in untreated (blue) and M3 (orange) treatment groups. (1) 3-methyl-2-oxovalerate, (2) 2-oxoisocaproate, (3) lactate, (4) threonine, (5) alanine, (6) acetate, (7) glutamate, (8) pyruvate, (9) citrate, (10) lysine, (11) creatine, (12) phosphocholine, (13) glycerophosphocholine, (14) glycine, (15) leucine, (16) valine, (17) isoleucine, (18) glucose.

[OFFICIAL]

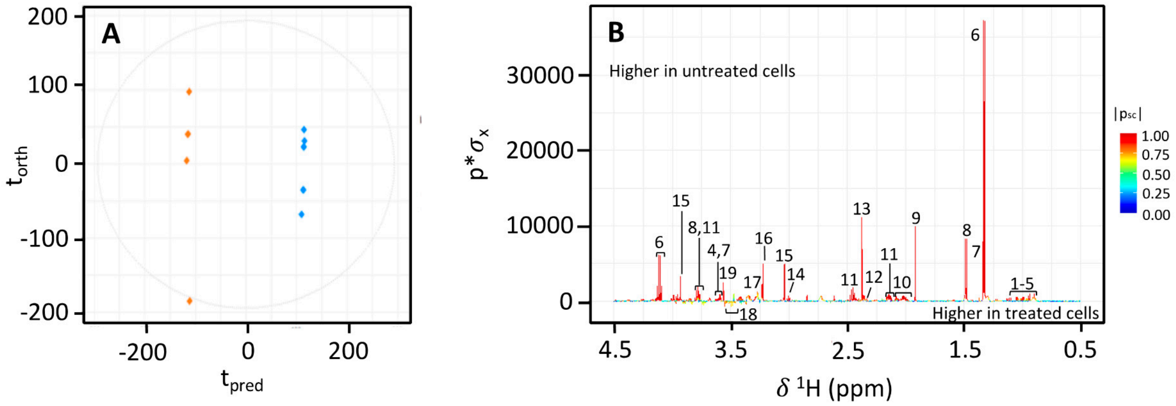

Figure S5. OPLS-DA scores plot (A, CV-AUROC=1.0) and corresponding coefficient loadings plot (B) derived from ^{1}H-NMR spectra of HepG2 cells treated with M6 for 48 h (orange) compared to untreated cells (blue). (1) 3-methyl-2-oxovalerate, (2) 2-oxoisocaproate, (3) leucine, (4) valine, (5) isoleucine, (6) lactate, (7) threonine, (8) alanine, (9) acetate, (10) proline, (11) glutamine, (12) glutamate, (13) pyruvate, (14) lysine, (15) creatine, (16) phosphocholine, (17) glycerophosphocholine, (18) glucose, (19) glycine.

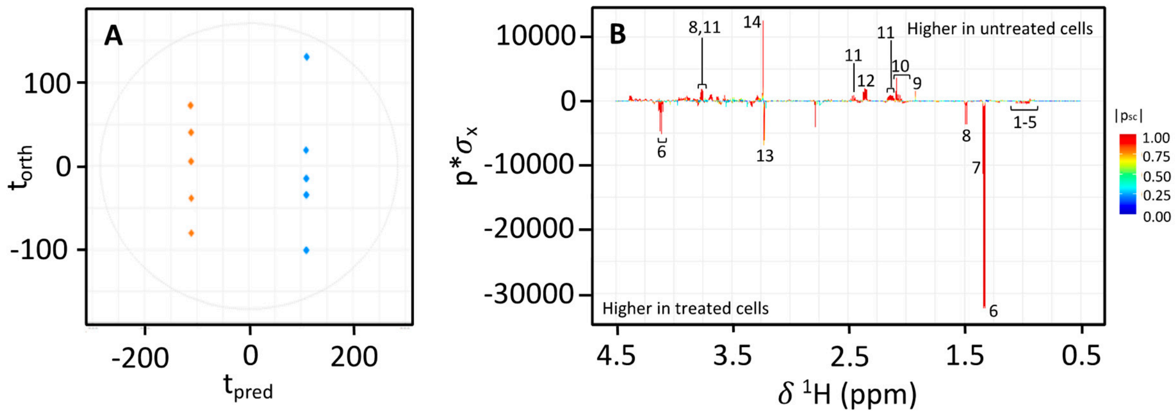

Figure S6. OPLS-DA scores plot (A, CV-AUROC=1.0) and corresponding coefficient loadings plot (B) derived from ^{1}H-NMR spectra of Caco-2 cells treated with M4 for 48 h (orange) compared to untreated cells (blue). (1) 3-methyl-2-oxovalerate, (2) 2-oxoisocaproate, (3) leucine, (4) valine, (5) isoleucine, (6) lactate, (7) threonine, (8) alanine, (9) acetate, (10) proline, (11) glutamine, (12) glutamate, (13) phosphocholine, (14) glycerophosphocholine.

[OFFICIAL]

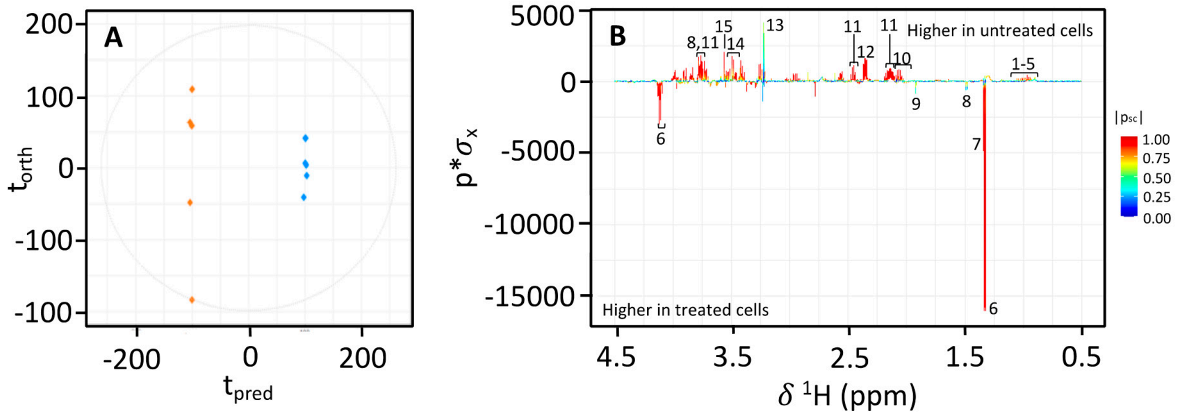

Figure S7. OPLS-DA scores plot (A, CV-AUROC=1.0) and corresponding coefficient loadings plot (B) derived from ^{1}H-NMR spectra of Caco-2 cells treated with M5 for 48 h (orange) compared to untreated cells (blue). (1) 3-methyl-2-oxovalerate, (2) 2-oxoisocaproate, (3) leucine, (4) valine, (5) isoleucine, (6) lactate, (7) threonine, (8) alanine, (9) acetate, (10) proline, (11) glutamine, (12) glutamate, (13) phosphocholine, (14) glucose, (15) glycine.

[OFFICIAL]
